# Ageing dynamics of ion bombardment induced self-organization processes

**DOI:** 10.1038/srep01850

**Published:** 2013-05-20

**Authors:** Oier Bikondoa, Dina Carbone, Virginie Chamard, Till Hartmut Metzger

**Affiliations:** 1 XMaS, The UK-CRG Beamline. European Synchrotron Radiation Facility. B.P. 220. F-38043 Grenoble cedex 09. France; 2 Department of Physics. University of Warwick. Gibbet Hill Road. Coventry, CV4 7AL, UK; 3 European Synchrotron Radiation Facility. B.P. 220. F-38043 Grenoble cedex 09. France; 4 Institut Fresnel. Aix-Marseille Université. CNRS. Ecole Centrale Marseille. Campus de Saint-Jérome, 13097 Marseille. France; 5 Max Planck Institute of Colloids and Interfaces. Department of Biomaterials. D-14424 Potsdam, Germany

## Abstract

Instabilities caused during the erosion of a surface by an ion beam can lead to the formation of self-organized patterns of nanostructures. Understanding the self-organization process requires not only the in-situ characterization of ensemble averaged properties but also probing the dynamics. This can be done with the use of coherent X-rays and analyzing the temporal correlations of the scattered intensity. Here, we show that the dynamics of a semiconductor surface nanopatterned by normal incidence ion beam sputtering are age-dependent and slow down with sputtering time. This work provides a novel insight into the erosion dynamics and opens new perspectives for the understanding of self-organization mechanisms.

Erosion is at the origin of remarkable examples of self-organization in nature such as the formation of evenly spaced valleys^1^ or large scale shoreline features^2^. Ripples and dunes are observed on sandy soils due to the action of wind on the sand bed^3^ and analogous structures can be formed also at the nanoscale by eroding surfaces with ion beams^4^. The relation between aeolian and ion induced patterns is not solely limited to a morphological resemblance: similar concepts serve to describe their dynamics. Indeed, continuum models for surfaces under ion beam sputtering (IBS) have been developed in close analogy to the hydrodynamic models used to describe the dynamics of granular matter. They explain quantitatively the interrupted pattern coarsening of a silicon surface bombarded at normal incidence with argon ions^5^. It is intriguing that very diverse non-equilibrium systems often display similar patterns.

Ion beam eroded surfaces represent a paradigmatic case of sustained non-equilibrium dynamics governed by the complex interplay between the mechanisms that tend to roughen and smoothen the surface^6^,^7^,^8^. The ion beam drives the surface out of equilibrium and dissipative mechanisms (atomic or defect migration, etc.) act to restore it. The time evolution of the surface morphology depends profoundly on control parameters such as the beam energy, angle of incidence of the ion beam on the surface, substrate temperature, etc.^8^. There is a significant amount of theoretical and experimental work reporting on the influence of the different control parameters on pattern formation by IBS (extensive bibliography can be found in Ref. 4). Some recent examples are focused on determining the instabilities leading to pattern formation^9^,^10^, stability/instability phase diagrams^11^, or the evolution of the surface morphology under different experimental conditions^12^,^13^,^14^. However, the dynamic properties of the patterns far from the instability threshold and the linear regime of exponential amplification have seldom been addressed. How does the local surface morphology of a IBS induced pattern evolve in time once the pattern is formed, far from the threshold and without changing any of the control parameters? Are the dynamics time-dependent or stationary?

To answer these questions, we propose to study the case of a well-documented model system, namely GaSb(001) sputtered at normal incidence. In 1999, Facsko *et al.* demonstrated that self-organized hexagonally ordered nanodot arrays can be created on a GaSb(001) surface by normal incidence ion beam sputtering^15^, a result that stimulated fundamental and applied research on IBS as an alternative method for the production of functional surfaces^16^. Normal incidence sputtering induces an initial transient smoothing of the GaSb(001) surface which is followed by roughening related to the formation of nanodots and the appearence of a self-organized pattern^17^ (Fig. 1(a)). For surfaces sputtered with ions in the 300–1200 eV energy range, the pattern wavelength (i.e. the mean distance between nanodots) coarsens until it reaches a saturation value. This behaviour, known as “interrupted coarsening”^18^, has also been found in other IBS nanopatterning experiments^5^,^19^. For GaSb(001), the wavelength saturation value scales with energy and the ordering of the pattern, as quantified by the lateral correlation length, is higher for lower ion energies^20^.

In the regime of wavelength saturation the ensemble average characteristics, such as the roughness or the average dot size and distance, may remain unchanged but the surface morphology is further evolving due to erosion. Experimental information about the dynamics in this regime can only be obtained via *in situ* and time-resolved measurements. X-ray scattering techniques offer the possibility of accessing statistical data about the morphology and crystalline structure of nanopatterned surfaces also *in situ* and with time resolution^19^,^21^. A grazing incidence small angle scattering (GISAXS) geometry allows to measure the scattered intensity distribution at low momentum transfer values, which is related to the lateral periodicity of a nanostructured surface^19^. However, one obtains information about the average morphology. Information about the time sequence of the local variations of the surface morphology, that is, the dynamics of the surface, can be obtained using the so-called “dynamic scattering” experiments^22^. Notably, under coherent illumination the scattered intensity recorded at low angles consists of a speckle pattern which depends on the exact surface morphology^23^. If the surface morphology varies with time, the corresponding speckle pattern will change accordingly and information about the underlying dynamics can be extracted from the speckle intensity fluctuations. This is the basis of the X-ray photon correlation spectroscopy (XPCS) technique^24^ which is the counterpart of the dynamic light scattering technique^25^ using coherent X-rays instead of laser light. We used XPCS in GISAXS geometry to access the surface dynamics in the pattern wavelength saturation regime of GaSb(001) surfaces during normal incidence sputtering.

## Results

**Figure 1:**
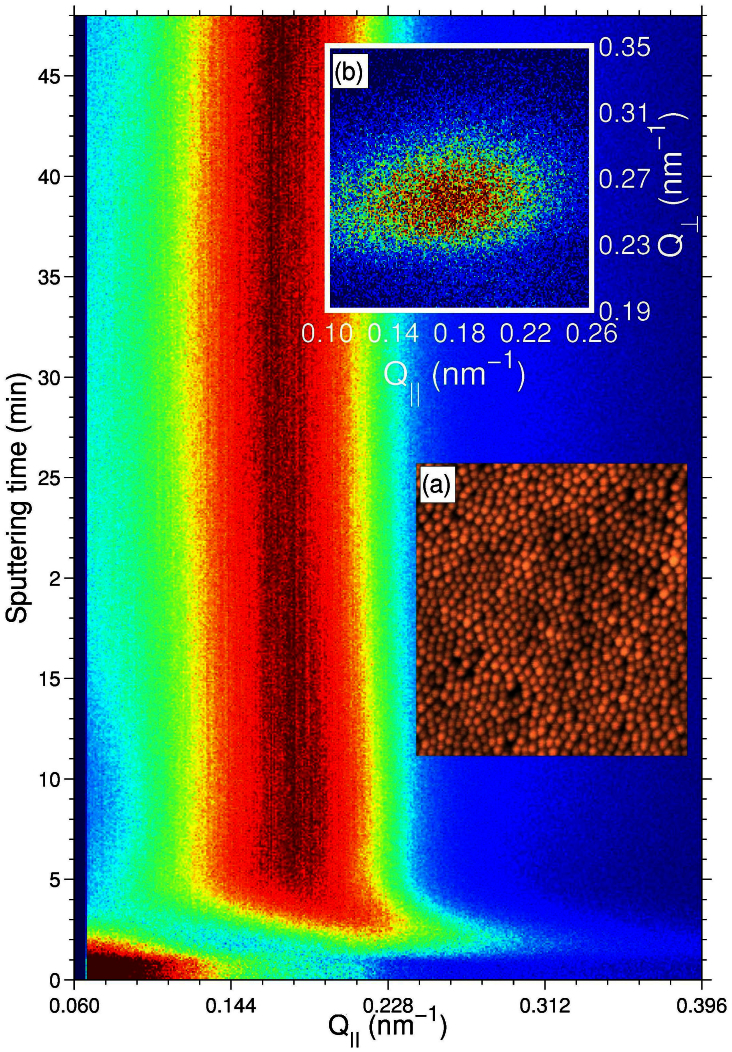
Coherent GISAXS: from direct space to Fourier space. GISAXS - time map for a GaSb surface eroded at 500 eV. The onset of a self-organized pattern is evidenced by the appearance of the correlation peak at *t* ~ 1 min (see text for details). Inset images: (a) Atomic force microscopy image (1 × 1 *μ*m^2^ area) of a GaSb sample sputtered at normal incidence. (b) The speckled correlation peak at t = 47 min.

**Figure 2:**
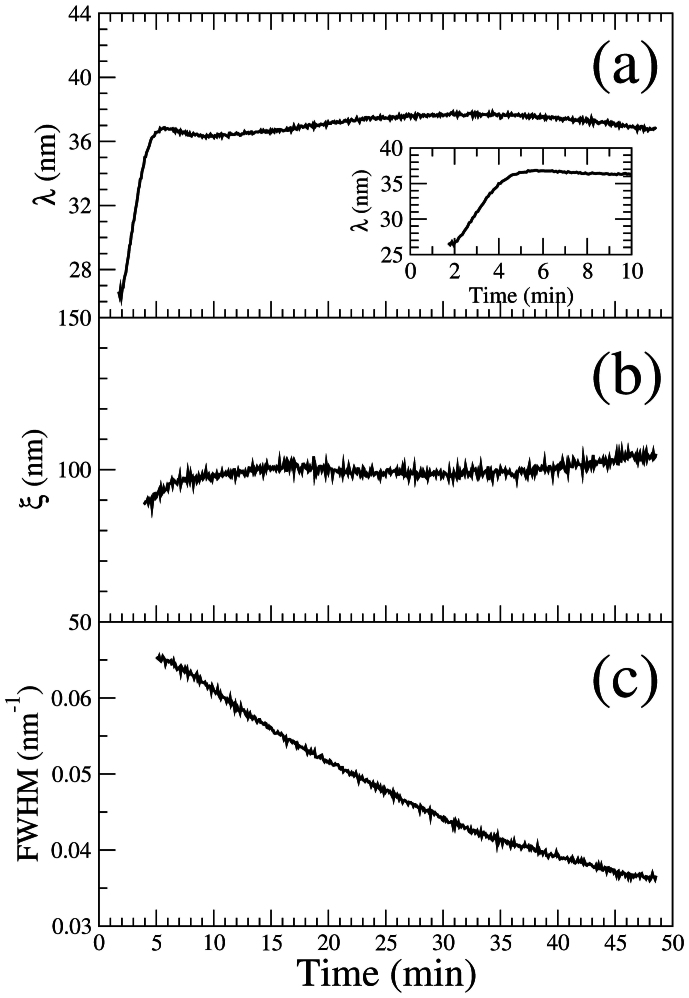
Time evolution of ensemble averaged characteristic quantities. Evolution vs. sputtering time of the: (a) pattern wavelength, *λ*. Inset: close-up of the wavelength coarsening regime; (b) correlation length, *ξ*; (c) Full width at half maximum (FWHM) of the correlation peak along *Q*_⊥_.

The sputtering process induces a progressive change of the surface morphology: the formation of ordered surface nanostructures with pattern wavelength (or lateral periodicity) *λ* is manifested in the GISAXS pattern by the development of order induced peaks at a specific Fourier component 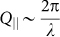. The inset (b) of Fig. 1 displays a typical CCD image showing a speckled correlation peak. The integrated peak intensity is shown in Fig. 1 as a function of the lateral momentum transfer direction *Q*_||_ and the sputtering time. At early sputtering times (*t* ≤ 1 min), diffuse off-specular scattering is present at low momentum transfer values due to surface roughness. In the *t* = 1–2 min range, a concomitant decrease of the diffuse signal and the appearance of a correlation peak are observed, evidencing the onset of the formation of a regular pattern on the surface. More information can be extracted from the position (*Q*_||_) and the width (Δ*Q*_||_) of the peak: the temporal evolution of the pattern wavelength 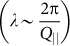 and of its lateral correlation length 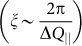 are shown in Fig. 2(a) & (b), respectively. Between *t* = 2–5 min, the pattern wavelength coarsens until it reaches a saturation value of *λ* ~ 36 nm. After saturation (*t* > 5 min), short lateral order with a normalized correlation length of *ξ*/*λ* ~ 3 is maintained during the whole sputtering time, in qualitative agreement with previous results^19^,^20^. Furthermore, the full width at half maximum (FWHM) of the correlation peak along the *Q*_⊥_ direction normal to the sample surface extracted from each CCD image decreases with time (Fig. 2(c)), indicating that the height of the nanodots increases with sputtering time, in agreement with the experimental results of Ref. 26.

**Figure 3:**
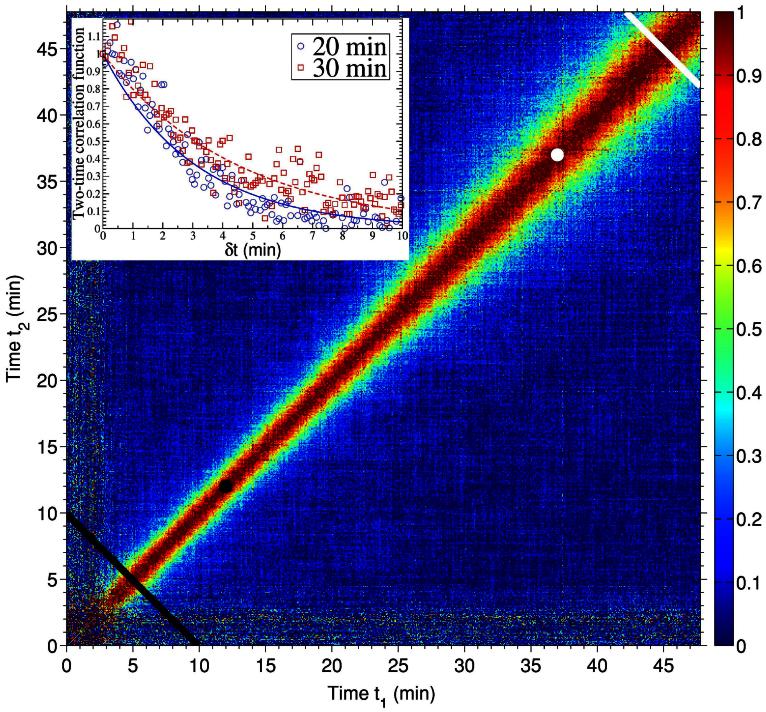
Two time correlation of the intensity fluctuations. Normalized two-time correlation function calculated for a rectangular region [Δ*Q*_||_ × Δ*Q*_⊥_ = (9.7 × 6.7) · 10^−3^ (Å^−1^)^2^] around the position of the correlation peak. The diagonal lines denote the start (black) and end (white) of the age range in which the one-time correlation functions have been extracted to calculate the correlation time. The change in the evolution of *τ_corr_* occurs at the ages 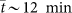 (black dot) and 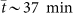 (white dot). The inset figure shows two cuts of the two-time correlation function at 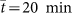 (blue open circles) and 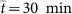 (red open squares) and the fits using a decaying exponential function (continuous lines).

Insight into the pattern dynamics during erosion in the regime of wavelength saturation is obtained from the time correlation of the speckle intensity fluctuations. In particular, we extract the two-time correlation function (TTCF)^27^,^28^, which, for non-equilibrium systems, has been proven particularly appropriate^29^. The resulting TTCF is shown in figure 3. It can conveniently be described by a set of two alternative variables: the sputtering time or sample age 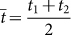 and the lag time *δt* = |*t*_1_ − *t*_2_|. Constant 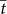 values corre spond to lines perpendicular to the *t*_1_ = *t*_2_ diagonal. The lag time is a measure of the distance from the diagonal^29^. The growing width of the signal along the diagonal of Fig. 3 evinces that the system depends on both, the lag time and the sample age. This is a usual behaviour in many non-equilibrium systems^30^. The dynamics are non-stationary and the speckle intensities are correlated for longer time as the sputtering time increases. For systems in equilibrium, the correlation time does not depend on the age, only on the lag time, and the stationary properties are studied using one-time (also called single-time) correlation functions^31^. For non-equilibrium systems, correlation times (*τ_corr_*) can be extracted from one-time correlation functions which are calculated for limited age intervals where the TTCF is quasi-stationary^32^.

**Figure 4:**
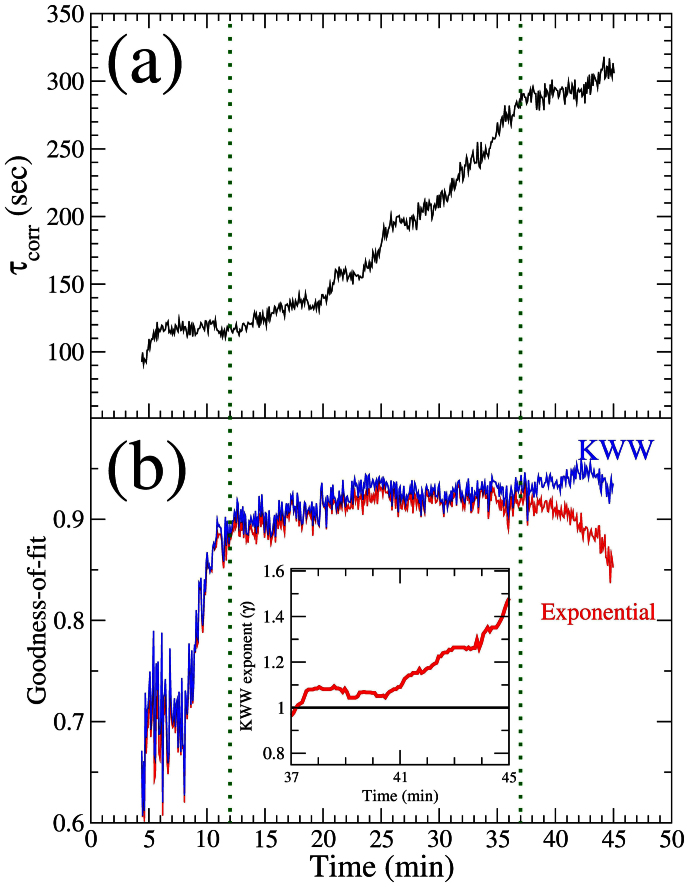
Quantitative values of the correlation time. (a) Evolution of the correlation time as a function of the sputtering time. (b) R-square goodness-of-fit using a single decaying exponential (red full line) and Kohlraush-Williams-Watts function (blue full line) to fit the correlation data. Inset: evolution of the KWW exponent for the late time regime. The vertical (green) dotted lines mark the separation between the three regimes of the dynamics (see text for details).

Three regimes can be distinguished in the evolution of *τ_corr_* (Fig.4(a)). In the early period (*t* = 5–12 min), the pattern wavelength has already reached the saturation value (see Fig. 2) and the correlation time is approximately constant, with *τ_corr_* ~ 119 s. For *t* = 12–37 min *τ_corr_* increases as *τ_corr_* ∝ *t*^1.6(1)^. This is distinctive of ageing: the dynamics become gradually slower with sputtering time. In these two regimes, the correlation function decays exponentially. Some short-lived transient behaviour is revealed by the slope changes on Fig. 4(a). At late stage (*t* > 37 min), the correlation time reaches a stable value (*τ_corr_* ~ 295 s) for about 6 minutes and starts to increase again. In this regime, as shown by the goodness-of-fit (Fig. 4(b)), the decay of the one-time correlation function is better described by a stretched exponential or Kohlrausch-Williams-Watts (KWW) function^33^,^34^ (see Methods). In the late regime, the KWW exponent *γ* evolves gradually from 1 to 1.5 (inset of Fig. 4(b)).

## Discussion

Numerous experimental studies have provided information about the basic spatial length scales of IBS induced self-organised patterns far from the threshold and their dependence on the different control parameters. However, less is known about the time scales involved. The case studied here corresponds to a pattern whose spatial characteristics are stable after saturation. Its lateral periodicity, characterised by the pattern wavelength, does not change during the whole sputtering time. However, the time evolution during the saturation is more complex. We have identified three regimes of the dynamics (Fig. 4). In the early regime (~ 5–12 min) the correlation time is approximately constant but the functional shape of the correlation function is still evolving. Most probably this is because at this stage the transition between the pattern wavelength coarsening and saturation regimes is not fully complete yet.

A slow down of the dynamics (ageing) is found in the second regime and the functional shape of the decay of the correlation is well reproduced by a decaying exponential. Ageing is a rather general behaviour that is observed in diverse systems such as glasses, granular media, growing interfaces, order-disorder phase transitions etc.^35^. It is also predicted in systems where competition between curvature-driven and an external-field driven interface motion occurs^36^, such as surfaces under IBS. Sputtering generates an amorphous layer that can be subjected to ion assisted viscous relaxation^37^ and therefore a glassy behaviour of eroded surfaces may be expected. In the case of glassy systems, a “cage-escape” scenario, where the motion of individual particles is caged by the neighbour particles, is the most accepted model. For that model, the characteristic correlation function decays are typically slower than exponential^38^. However, our results demonstrate that the ageing is not due to a lateral rearrangement of the nanodots during IBS. We have measured either exponential or faster decays which rules out a “cage-escape” behaviour. We can also discard that the system is subjected to coarsening-type dynamics. In coarsening systems, the size of the ordered regions grows with time and ageing arises due to a slowing down of the motion of domain boundaries^39^. But we observe that in the wavelength saturation regime the average surface domain width remains unchanged (Fig. 2(b)), implying that the average area of the ordered regions and their boundaries do not change appreciably and therefore are not the cause of the ageing. We attribute the ageing to the gradual increase of the nanodot height. As shown in Fig. 2(c), the FWHM of the correlation peak along *Q*_⊥_ decreases with time, showing that the height of the nanodots continues to increase with the sputtering. The effect of the nanodot height increase on the dynamics is two-fold: (1) As the nanodots grow higher, it takes more time to the system to decorrelate from earlier configurations. In other words, the dot creation/annihilation rate decreases and the patterns are preserved for longer times; (2) Mass redistribution, which is a crucial process controlling pattern formation^10^,^11^,^40^,^41^,^42^,^43^,^44^, is hindered. With higher dots and a more abrupt topography, the flow of material is also altered and this may result in a slowing down of the pattern dynamics.

The progressive change of the functional shape of the correlation function from an exponential to a faster-than exponential decay that occurs at *t* > 37 min reveals that processes with different timescales are involved in the dynamics^45^. The transition to this regime is probably due to a building up of stress at the surface. Stress is induced during IBS and, as it has been pointed out, may actually play a prominent role during pattern formation^46^. A similar KWW exponent (*γ* ~ 1.5) has been found for diverse systems (polymeric systems, colloids in glassy solvents, etc.) and related to stress relaxation mechanisms^38^,^47^. Notably, stress relaxation has also been suggested as the cause of the ageing behaviour found in granular media^48^, which has many similarities with surfaces under IBS^43^.

In summary, we have determined from XPCS data that under ion beam bombardment and without any change of the sputtering conditions, the dynamics of nanodot formation on GaSb(001) in the pattern wavelength saturation regime are not stationary, evolve with time and show ageing. The ageing is related to the growth in height of the nanodots which reduces the nanodot creation/anihilation rate and results in more persistent arrays. Furthermore, the increase of stress at the surface caused by sputtering probably modifies the dynamics of nanopatterning. Our results experimentally corroborate that measurements of the correlation functions using XPCS provide new insights into the self-organization dynamics that cannot be obtained from measurements of the pattern wavelength or roughness evolution alone, as we have recently proposed based on calculations^49^. They also open new prospects of studying other important surface phenomena such as, for example, the evolution of ripples during off-normal sputtering. A general question that arises is whether ageing exists in granular systems in which coarsening also ceases, e.g. in the formation of giant sand dunes^50^. Besides their intrinsic interest for the understanding of nanopattern formation in nonequilibrium systems, these result are also relevant for the improvement of fabrication processes relying on self-organization mechanisms.

## Methods

### Data acquisition

The experiments were carried out at the ID01 beamline of the European Synchrotron Radiation Facility (ESRF). The GaSb(001) samples were mounted inside a vacuum chamber especially adapted for coherent scattering experiments^51^. A channelcut Si(111) monochromator was used to select 8 keV (1.55 Å wavelength) energy photons from the radiation generated with an undulator X-ray source. 10 × 10 *μ*m^2^ slits were used to select a transversely coherent beam. At this energy, the flux on the sample is ~ 5 × 10^9^ photons/s. A Princeton charge-coupled device (CCD) with 20 × 20 *μm* pixel size and 1340 × 1300 pixels, placed at 1.38 m from the sample, was used to record the scattered intensity at the first correlation peak. The GaSb(001) sample (~ 10 × 10 × 1 mm) was mounted on a copper plate and glued using a small drop of silver paint underneath. The sample was sputtered at normal incidence using 500 eV argon ions delivered by a Kaufman-type ion source from *Veeco Instruments*. The ion beam was not neutralised but we cannot rule out the exposure of the sample to any other material present in the sputtering chamber via secondary collisions. The ion flux on the sample was ~ 3.75 · 10^14^ *ions*/*cm*^2^. The sample temperature did not exceed 50°C and the argon pressure was kept constant at a value ~ 1.25 · 10^−3^ mbar. Further details about the chamber and the ion source can be found in Ref. 51.

The images were acquired during sputtering in a continuous mode, i. e., with no interruption of the erosion process, at a rate of one frame per 4.7 seconds for 1 second exposure. The total sputtering time was 48 minutes. The CCD images were subsequently treated using the droplet algorithm to suppress the electronic noise^52^. We carefully checked by continuously monitoring the incoming X-ray beam intensity and the ion source and sputtering parameters (i.e. filament current, beam and accelerator current, pressure and sample temperature) that there were no factors such as sample or intensity drifts that could potentially alter the results about the dynamics. Finally, the reproducibility of the observed features was also verified by repeating XPCS measurements on another sample. We also checked, using atomic force microscopy, that the morphology of the samples used during the XPCS experiments and other samples prepared offline in the same conditions were similar.

### Calculation of the two-time correlation function

The normalized intensity fluctuations are given by: 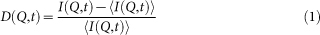
where 〈*I*(*Q*,*t*)〉 is the average intensity that is measured in a conventional X-ray diffraction experiment where the spatial and temporal average over the sample is obtained^53^. For non-equilibrium systems such as the particular case of surfaces under ion bombardment, 〈*I*(*Q*,*t*)〉 changes with time even when the saturation of *λ* has been reached, as the lateral ordering may further increase^20^. We calculated 〈*I*(*Q*,*t*)〉 following the procedure described in Ref. 28.

The two-time correlation function (TTCF) is obtained from the average of the product of the speckle intensity fluctuations: 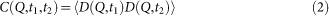
where the average is done over pixels corresponding to a range of Δ*Q* in which the correlations are not expected to change significantly. The averaged diagonal values have been used to normalize the TTCF^27^. The more usual one-time correlation function can be obtained from the TTCF by summing up the TTCF at fixed lag time, *δt* = *t*_2_ − *t*_1_^47^: 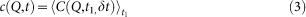
One-time correlation functions were extracted for different ages by averaging the TTCF over age intervals of 2Δ*t*. The one-time correlation functions have been fitted using a single exponential 
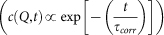
 and a stretched exponential or Kohlrausch-Williams-Watts (KWW) function^33^,^34^, characterized by the KWW *γ* exponent in the decay function 
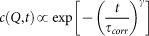
, *τ_corr_* being the correlation time. We have used a R-square statistics for the goodness-of-fit. R-square denotes the proportion of the variance explained by the fit. The closer its value is to 1, the better the fit is.

## Author Contributions

O.B., D.C. and T.H.M. devised the study. O.B. and D.C. prepared the setup for coherence experiments. O.B. analysed the data and wrote the necessary code. D.C. and V.C. helped with the treatment and interpretation of coherent data. All authors participated in the experiments and discussed the results. O.B. wrote the manuscript with contributions from all.
